# Impact of COVID-19 on adolescents’ mental health: a systematic review

**DOI:** 10.1186/s43045-020-00075-4

**Published:** 2020-12-22

**Authors:** Gilbert Sterling Octavius, Felicia Rusdi Silviani, Alicya Lesmandjaja, Angelina, Andry Juliansen

**Affiliations:** 1 Faculty of Medicine, University of Pelita Harapan, Karawaci, Tangerang, Banten Indonesia; 2 Department of Pediatric, Faculty of Medicine, University of Pelita Harapan, Karawaci, Tangerang, Banten Indonesia

**Keywords:** COVID-19, Mental health, Adolescent, Depression, Anxiety

## Abstract

**Background:**

The impact of COVID-19 towards psychology and mental health is anticipated to be significant and may affect the population disproportionately, especially adolescent as the vulnerable category. We aimed to analyze the impact of COVID-19 towards adolescents’ mental health.

**Main body:**

A systematic search was conducted from Cochrane, Google Scholar, Scielo, and PubMed. Inclusion criteria included all types of studies which observed the effect of COVID-19 and its related causes, such as lockdown, on adolescents’ mental health. All studies were assessed for its level of evidence according to Oxford 2011 criteria and Newcastle Ottawa Scale (NOS). Three studies (Seçer and Ulaş, Int J Ment Health Addict: 1–14, 2020; Zhou et al., Eur Child Adolesc Psychiatry 29:749–58, 2020; Qu et al., Lancet: 1–17, 2020) showed that COVID-19 was a risk factor for mental health problems in adolescents while Oosterhoff et al. (J Adolesc Health 67: 179–185, 2020) showed that adolescents who preferred to stay at home during this pandemic reported less anxiety and depressive symptoms

**Conclusion:**

COVID-19 has been found to be associated with mental health changes in adolescents which meant management of COVID-19 should also focus on mental health as well.

## Background

Coronavirus disease-19 (COVID-19) was found initially in Wuhan, Hubei Province, China, on December 31, 2019, and it continues to be a pandemic [1]. Until July 13, 2020, it has infected about 12,750,275 people and caused 566,355 deaths around the world while showing no signs of slowing down [2]. Based on this fact, many countries around the world had applied physical distancing and closed public places such as schools, campuses, offices, and public places to curb the transmission [3–6]. On the other hand, physical distancing has impacted mental health by depriving social contact, especially the adolescent [1, 7, 8]. Adolescent is defined as individuals in the 10–19 years age group [9] in which it is a vulnerable age group to develop negative mental health impairment because they are very sensitive to psychological and social transformation. Adolescent experiences higher peer interaction and social world than with their family, and even forms complex peer relationship compared to their younger counterparts such as babies and children. Any separation from peer relationships such as rejection, bullying, or loneliness has been linked to mental health disorder such as depression, anger, fear, stress, and anxiety [10]. Physical distancing had led individuals to cut off social interaction unintentionally because individuals had the tendencies to avoid conversation in order to limit meetings. Although most adolescents were exposed to physical distancing, lockdown, or quarantine during this pandemic of COVID-19, the adolescents’ mental health changes can be variative based on their circumstances and motivation to obey physical distancing [11]. This study aims to analyze the impact of COVID-19 towards adolescent’s mental health, with the hypothesis that COVID-19 is associated with poor mental health outcomes on adolescents.

## Methods

This systematic review was conducted based on the Preferred Reporting Items for Systematic Review and Meta-Analysis (PRISMA) statement [12]. The protocol of this systematic review has been registered in The International Prospective Register of Systematic Reviews (PROSPERO) database (CRD42020195764).

We included cohort studies and cross-sectional studies which observed the effect of COVID-19 and its related causes such as lockdown on adolescents’ mental health impact such as depression, fear, and anger. Exclusion criteria comprise of studies that include correspondents with age group extending below and over the adolescents’ age range (10–19 years old), as well as studies which include adolescents with pre-existing mental health problems such as autism spectrum disorder (ASD) or post-traumatic stress disorder (PTSD).

A systematic literature search was performed on July 7, 2020, using four different databases such as Cochrane, Google Scholar, Scielo, and PubMed using keywords listed in Table 1. Literature selection was performed from 2019 onwards and restricted to only publications that are in English language.

**Table 1:** Keywords used in each search engine

Search engine	Keywords
PubMed	((((((((“COVID-19”[Title] OR “COVID-2019”[Title]) OR “severe acute respiratory syndrome coronavirus 2”[Title]) OR “2019-nCoV”[Title]) OR “SARS-CoV2”[Title]) OR “2019nCoV”[Title]) OR “Wuhan”[Title]) OR “coronavirus”[Title]) AND “Adolescent”[Title]) AND (“psychological impact”[Title] OR “mental health”[Title])
Scielo	((((ti:(covid-19))) OR ((ti:(covid-2019))) OR ((ti:(severe acute respiratory syndrome coronavirus 2))) OR ((ti:(2019-ncov))) OR ((ti:(sars-cov2))) OR ((ti:(wuhan))) OR ((ti:(2019ncov)))) OR (ti:(coronavirus))) AND ((ti:(adolescent))) AND ((ti:(psychological impact)) OR (ti:(mental health)))
Cochrane	(covid-19 OR covid-2019 OR severe acute respiratory syndrome coronavirus 2 OR 2019-ncov OR sars-cov2 OR wuhan OR 2019ncov OR coronavirus) AND (adolescent) AND (psychological impact OR mental health)
Google Scholar	COVID-19 AND Psychological Impact AND Adolescent AND Mental Health

Data from each study was extracted in a standardized form, compiling study citations, baseline characteristics of the included subjects, and the study findings. Study citations included the name of the first author, year of publication, and title of the study. Meanwhile, characteristics of each study refered to study design, location of the study, and patients’ characteristics (age, ethnicity, gender, sample size, and family income). The study findings extracted involved the odds ratio, *R*^2^, or *β* value analyzed in each study.

Three independent reviewers conducted the quality assessment of the studies (GS, FR, AL). The included studies were critically appraised using Newcastle Ottawa Quality Assessment Scale (NOS) for case control and cohort studies [13]. Any discrepancies of NOS score between reviewers were discussed until it reached a conclusion. If the discrepancies are still not settled, two expert reviewers (A, AJ) were consulted, and decisions were made by them. High-quality studies were defined as studies fulfilling NOS score of minimum 7 (Table 2). Data was synthesized based on a minimum of four different and high-quality studies with consistent finding. The obtained data was analyzed considering the method of variable analysis used, study size, odds/hazard ratio, along with its confidence interval.

**Table 2:** Quality assesment of the included studies using Newcastle Ottawa Scale

Type of study	Selection	Comparability	Outcome	Total
Oosterhoff et al. [15]	****	*	**	7
Seçer and Ulaş [16]	****	*	**	7
Zhou et al. [11]	****	*	***	8
Qu et al. [14]	****	*	**	7

Each * represents a point for fulfilling the NOS criteria

## Results

**Fig. 1:**
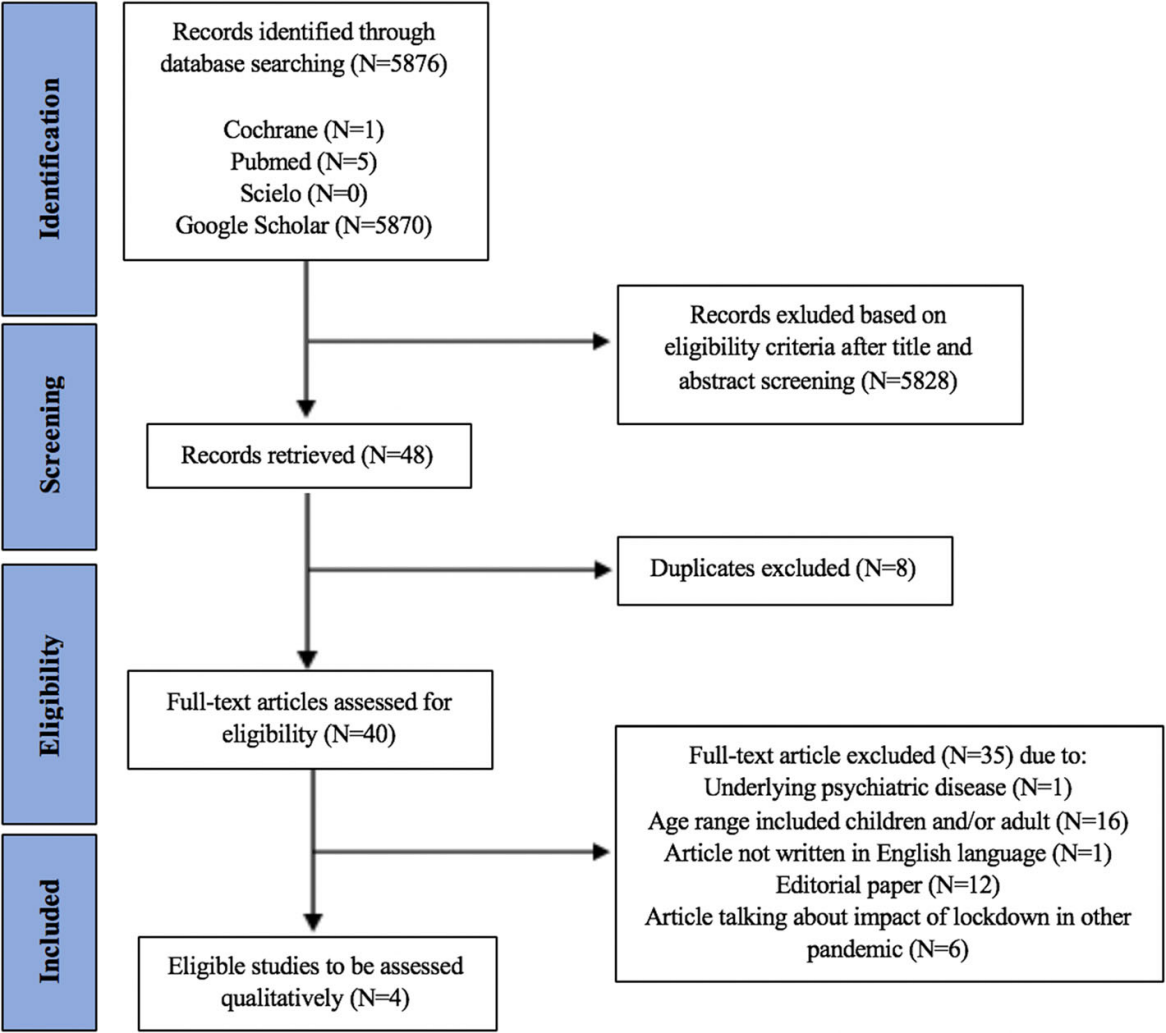
PRISMA flowchart of study selection

Literature search was done using keywords listed in Table 1. Out of 5876 articles identified, 48 articles were retrieved after title and abstract screening. Duplicates were then removed, and after ensuring the remaining articles were appropriate according to the writers’ inclusion and exclusion criteria, four articles were chosen for this study which were studies by Qu et al. [14], Oosterhoff et al. [15], Zhou et al. [11], and Seçer and Ulaş [16]. The flow of our study selection is presented in Fig. 1 according to the PRISMA statement [12].

The summary of baseline study characteristics is presented in Table 3. All studies had level 2 of evidence based on Oxford 2011 and were considered high-quality based on each NOS score (Table 2). All studies were cross-sectional studies except for Qu et al. [14], which was a prospective study, which means that temporality or causality cannot be established. Each study also used different questionanires to assess pyschosocial impact and mental health except Zhou et al. [11] and Qu et al. [14] which both used Patient Health Questionnaire (PHQ-9) and Generalized Anxiety Disorder Scale (GAD-7) to assess depression and anxiety, respectively. All of the questionnaires are self-administered online except in the study of Qu et al. [14] which initially was administered by trained staffs.

**Table 3:** Summary of baseline characteristics and outcomes of included studies

Authors	Type of study	Study location	Age group (mean ± SD)	Sample size		Outcomes studied		Findings (OR/*β*/*R*^2^ to develop adverse mental health)
				Total	M/F (%)	Questionnaire used	Component assessed	
Oosterhoff et al. [15]	Cross-sectional	USA	13–18 (16.35 ± 1.13)	683	22.7/75.3	1) Patient-Reported Outcomes Measurement Information System (PROMIS) anxiety scale 2) PROMIS depression scale 3) Interpersonal Needs Questionnaire	1) Anxiety 2) Depression 3) Belongingness and burdensomeness	No association between degree of social distancing engagement and any indicator of mental or social health across all models (*R*^2^ adjusted for anxiety 0.083; − 0.2–0.15; *p* value 0.09), (*R*^2^ adjusted for depression 0.057; − 0.28–0.05; *p* value 0.09), (*R*^2^ adjusted for burdensomeness 0.02; − 0.27–0.27; *p* value 0.14), and (*R*^2^ adjusted for belongingness 0.12; − 0.01–0.47; *p* value 0.12)
Seçer and Ulaş [16]	Cross-sectional	Turkey	14–18 (16.4 ± 2.14)	598	38.9/61.1	1) Obsessive Compulsory Inventory-Child Version 2) Emotional Reactivity Scale 3) Depression and Anxiety Scale for Children 4) The Fear of COVID-19 Scale 5) Experiential Avoidance Questionnaire	1) Obsessive compulsive symptoms 2) Emotional response 3) Depression and anxiety symptoms 4) Depression and anxiety symptoms due to COVID-19 5) Avoidance responses against various experiences	Fear of COVID-19 positively predicts emotional reactivity (*β* = .50, *p* < .01), and emotional reactivity positively predicts experiential avoidance (*β* = .59, *p* < .01) and depression-anxiety (*β* = .81, *p* < .01).
Zhou et al. [11]	Cross-sectional	China	12–18 (N.A)	8079	46.5/53.5	1) Patient Health Questionnaire (PHQ-9) 2) Generalized Anxiety Disorder Scale (GAD-7)	1) Depression 2) Anxiety	Female gender (DE 1.15; 1.05–1.26) (AN 1.1; 1.001–1.21) (*p* = 0.048), being in Hubei province (DE 1.58, 1.34–1.87) (AN 1.64; 1.39–1.93) (*p* < 0.001), and being in junior grade three (DE 1.4; 1.11–1.75) (AN 1.32; 1.04–1.67) (*p* < 0.001) confers higher risk factor for depressive and anxiety symptoms meanwhile awareness of COVID-19 was protective against depressive and anxiety symptoms
Qu et al. [14]	Prospective cohort	China	12–17 (14.33 ± 1.12)	14,241	51.11/48.89	1) Patient Health Questionnaire (PHQ-9) 2) Generalized Anxiety Disorder Scale (GAD-7) 3) Childhood Trauma Questionnaire (CTQ-SF) 4) Connor-Davidson resilience Scale (CD-RISC) 5) Propensity Score Matching	1) Depression 2) Anxiety 3) Childhood abuse 4) Resilience 5) Probability of exposure risk	Depression 2.3 (1.7–3.1; *p* < 0.0001) and anxiety 2.1 (1.6–2.8; *p* < 0.0001) in adolescents with an exposure risk.

*DE* Depression, *AN* Anxiety

The study done by Zhou et al. [11] showed that prevalence of mild-to-severe depressive and anxiety symptoms in Chinese adolescents during COVID-19 outbreak was 43.7% and 37.4% respectively. The prevalence of adolescents with both depressive and anxiety symptoms was 31.3%. They found that adolescents living in cities were less depressed (37.7%). Zhou et al. [11] also found that adolescents living in cities were less likely to have depressive or anxiety symptoms (37.7% vs 47.5% and 32.5% vs 40.4%). The scores in COVID-19 knowledge, prevention, and control measures, and projections of COVID-19 were higher in adolescents without depressive and or anxiety symptoms. Utilizing multivariate logistic regression analysis, Zhou et al. [11] found that female gender (OR_(Depression[DE])_ = 1.15, 1.05–1.26; OR_(Anxiety[AN])_ = 1.10, 1.00–1.21), living in Hubei province (OR_DE_ = 1.58, 1.34–1.87; OR_AN_ = 1.64, 1.39–1.93), and being in the junior grade three (OR_DE_ = 1.40, 1.11–1.75; OR_AN_ = 1.32, 1.04–1.67) were risk factors for depressive and anxiety symptoms.

The study done by Qu et al. [14] is the only study included to be using prospective cohort as its study design. First, the authors did the first survey round as a baseline data which assessed their background factors, depression, anxiety, resilience, and childhood maltreatment using the questionnaires listed in Table 3. The second round of survey was done 3 months after the first round of data gathering, and after excluding invalid questionnaires the valid follow-up rate was 74.91% and varied by region (*p* < 0.001). They found that just before the pandemic, 51.51% of adolescents reported depressive symptoms and 38.53% reported anxiety symptoms. After home confinement, the number dropped to 38.29% and 23.73% respectively (all *p* < 0.0001). After Propensity Score Matching (PSM) matching, adolescents with exposure risk still had more depression (60.54%; *p* = 0.0023) and anxiety symptoms (41.26%; *p* = 0.0072) than those without any exposure risks (45.95% and 28.83% respectively). Exposure risk was defined as anyone in the surrounding living environment of the participant who was infected with COVID-19. Qu et al. concluded that adolescents with exposure risks (assessed with assessed with PSM analysis [PSM] analysis) are 2.3 times more likely to suffer from depression (1.7–3.1; *p* < 0.0001) and 2.1 times more likely to suffer from anxiety symptoms (1.6–2.8; *p* < 0.0001) in adolescents with exposure risk.

Using standardized structural equation modeling (SEM), Seçer and Ulaş [16] found that fear of COVID-19 positively predicts emotional reactivity (*β* = .50, *p* < 0.01) which positively predicts experiential avoidance (*β* = .59, *p* < 0.01) and depression-anxiety (*β* = .81, *p* < 0.01). Oosterhoff et al. [15] did not find any association between degree of social distancing and any indicator of mental or social health. This study also found that those who were social distancing because of social responsibility reported less anxiety symptoms (*R*^2^ = 0.03, standard error (SE) = 0.12 [− 0.21, 0.27]), depressive symptoms (*R*^2^ = − 0.05, SE = 0.12 [− 0.29, 0.19]), and burdensomeness (*R*^2^ = − 0.35, SE = 0.19 [− 0.73, 0.03]) (*p* < 0.05) which was also found in adolescents who would have preferred to stay at home (*R*^2^_anxiety_ = − 0.3, SE = 0.11 [− 0.51, − 0.09], *R*^2^_depressive_ = − 0.35, SE = 0.1 [− 0.55, − 0.14], and *R*^2^_belongingness_ = − 0.03, SE = 0.16 [− 0.29, 0.35) (*p* < 0.05). Additionally, adolescents who were social distancing because a friend told them they should reported greater depressive symptoms (*R*^2^ = 0.26, SE = 0.13 [0.02, 0.51]) (*p* < 0.05) while adolescents who were social distancing because they wanted to avoid judgment reported greater anxiety symptoms (*R*^2^ = 0.35, SE = 0.17 [0.03,0.68]) (*p* < 0.05).

## Discussion

COVID-19 is an unprecedented infectious disease which has taken a massive toll on deaths. Sudden changes in lifestyle, including lockdown, quarantine, or physical distancing coupled with the loss of loved ones, may take a toll on mental and emotional health. Although there has been several studies in the past trying to elucidate how quarantine and isolation affect mental health, the extent on how far adolescents’ mental health is affected is unknown [17]. In this systematic review, three included studies show that COVID-19 impacts on adolescents’ mental health with one study citing that there was no significant impact.

The cohort result from Qu et al. [14] should be interpreted carefully. There was 25.09% drop-out from the participants in the first round to the second round, so the result from first and second round might not be able to be compared directly. The study also found that the incidence and severity of anxiety and depressive symptoms dropped significantly after home confinement. It meant that mental health was not affected in overall adolescent population, but only in exposure risk population. Furthermore, there was only 2.2% subjects that reported a risk of exposure. As this study analyzed COVID-19 exposure risk as independent variable, the low number of subjects in exposure risk might not be able to represent a real general population. Despite its limitations, this study had a baseline data to compare pre-home confinement and intra-home confinement, which made it one of the best quality studies among the others.

Zhou et al. [11] presented a similar finding with Qu et al. [14] that living in community with high number of COVID-19 cases was a risk factor towards depression and anxiety. These two studies also used same questionnaires to assess depressive and anxiety symptoms. However, Zhou et al. [11] found that awareness of COVID-19 was a protective factor against depressive and anxiety symptoms while Qu et al. [14] stated that some protective factors, such as good parent-child relationship, few adverse experiences, good family structure, and high resilience could be outweighed by exposure of COVID-19. These different results showed that there were a lot of factors that might affect the rise of depressive and anxiety symptoms in adolescent in COVID-19 pandemic era.

Study by Seçer and Ulaş was unique because it is the only study which assessed obsessive-compulsive disorder (OCD) due to fear of COVID-19 [16]. The reasoning behind it was due to the fear of this disease, adolescents will start a washing and hoarding obsession. Although the study did not mention the number of positive cases in the area, the common denominator of fear of COVID-19 is still maintained in this study. Seçer and Ulaş [16] went a step further by theorizing that emotional reactivity might be one of the various psychopathologies that might explain depression, anxiety, and OCD in adolescents while experiential avoidance mediates fear of COVID-19 and OCD symptoms. However, while trying to establish a causality, this paper is a cross-sectional study which means that further studies need to consider other study designs in order to establish the direct link between fear of COVID-19 with OCD symptoms, if possible.

Although the study done by Oosterhoff et al. [15] found that there was no association between degree of social distancing and mental or social health, this study found that motivations for social distancing were differentially associated with degree of social distancing as well as depressive symptoms, anxiety symptoms, burdensomeness, and belongingness. Adolescents who were social distancing because they wanted to avoid social judgment reported more anxiety symptoms which might be explained by the fact that past researches shows that symptomatic youths are more sensitive towards social judgment or peer rejection [18]. This study also found that adolescents who prefer to stay at home reported lower anxiety and depressive symptoms because adolescents who chose to stay at home might be struggling less with reduced social contact [15]. However, some motivations studied have low samples such as “no alternatives” (17.8%), “friends said I should” (13.9%), “avoid judgment” (7%), and others (4.4%) which could affect the significancy of the associations studied. High compliance towards social distancing in the sample population could be seen from the motivations of social responsibility (78.1%) and not wanting others to get sick (77.9%). It implies that the adolescents in the population studied have a good knowledge of COVID-19 which confers protective factor towards depression and anxiety just like in the study done by Zhou et al. [11].

Onset of depression is affected by both genetic factors and external environmental factors such as stressful life events [19, 20]. Adolescents are especially vulnerable to stressful life events and could lead to lower levels of motivation, lower concentration, poorer achivement [21], psychological distress, anxiety, depression [22], and suicide [23]. In fact, mental health conditions account for 16% of the global burden of disease and injury in people aged 10–19 years old and half of all mental health conditions starting at 14 years old, but most cases were undetected and untreated [24]. Moreover, non-emergency medical services were halted or redirected towards more emergency cases and hence medical care for affected adolescents will be affected [25]. Fear of COVID-19 significantly increased negative affect, anxiety, and depression [26]. Qu et al. [14] stated that long-term confinement had no adverse mental health impact on adolescents from regions with a low incidence of COVID-19. Compared to Qu et al. [14], the exposure risk of COVID-19, living area, and fear of COVID-19 which could be the risk factors for mental health changes on adolescents are not measured in Oosterhoff et al.’s study [15]. A study done by Fitzpatrick stated that the fear of COVID-19 is not distributed uniformly across the USA, and there were specific concentrated COVID-19 fear in more crowded populated communities, communities with higher presumptive and reported cases of COVID-19, and urban locations [27]. Most studies were also originated from high-income countries, which may affect the generalizability of the findings to low-income and middle-income countries which are often under-presented in terms of generating evidences through empirical studies [28]. Therefore, the result which was conducted in high income countries such as the Oosterhoff’s [15] study cannot be generalized to other low- and middle-income countries that were exposed to COVID-19.

Adolescents who are infected by COVID-19 would also need psychiatric care as 10% of infected children who experienced trauma due to infection and its consequences might be diagnosed with PTSD [29]. Psychiatrists and pediatricians need to be aware of warning signs of mental health problems in COVID-19 infected children such as mood swings and psychosis-like symptoms as psychiatrists need to work directly with young patients and their families in order to screen and detect mental disorders as soon as possible with adequate personal protective equipment [25].

Zhou et al.’s [11] study suggested that the level of knowledge, prevention, and control measures for COVID-19 are protective factors against the development of depressive and anxiety symptoms. However, there are media/press sources that might give false information and reports about COVID-19 which could lead to anxiety and depressive symptoms of the public. A study by Zhong et al. also suggested that health education aimed at increasing knowledge of COVID-19 are important to keep optimistic attitudes [30]. This is related to a statement from Zhou’s study that said positive and optimistic attitudes towards COVID-19 epidemic’s development are also protective factors against depressive and anxiety symptoms [11].

The government and health authorities’ role are important in COVID-19 crisis to provide adequate information and deny any false information to keep the public informed. In crisis situation like this, it is convenient to educate and provide public with the accurate information through digital media platform. A study by Liu suggested that the uses of digital media might initiate preventive behaviors directly or indirectly. It states that seeking information about COVID-19 from online news media, media social networking (MSNs), social live streaming services (SLSSs) was associated with increased preventive practices [31]. Since there are a lot of misinterpreted informations from the internet, it is important to obtain information from a credible website such as the national Centers for Disease Control (CDC), World Health Organization (WHO), or from other sources endorsed by these authorities, rather than a general search on the internet or social media [32].

A rapid systematic review done by Imran et al. found that quarantine is associated with significant negative impact on mental health of children and adolescents which might persist for months or years after the quarantine. Although the cause of quarantine is very diverse (from natural disasters such as Tsunami in Aceh 2004 to children requiring ventilators at home), this study proposed some interventions that could be done to reduce the impact of mental health during quarantine such as provision of psychosocial support, dissemination of accurate information, limit exposure to news, positive parenting, social connectivity, as well as behavior activation complemented with sleep hygiene, exercise, and healthy eating [33]. Another systematic review done by Loades et al. [34] which assessed the impact of social isolation and loneliness due to COVID-19 on children’s and adolescent’s mental health found that children and adolescents are more prone towards high rates of depression and anxiety during and after enforced isolation ends which might last up to 0.25 to 9 years later.

Our study is limited by exclusion of articles written in languages other than English as well as unexplored gray literatures. The studies included also did not measure any baseline characteristics except for study done by Qu et al. [14]. Moreover, this systematic review could not be proceeded to meta-analysis due to the heterogenous use of variable analysis. Therefore, we recommend future studies to use a more homogenous questionnaire as well as their odds ratio presentation clearly to depict the effect of COVID-19’s mental health impact in adolescents. Lastly, correlation between those who were at low risk of contracting COVID-19 and the lesser impact on mental health risk may be independent of the actual risk and was just based on individual’s subjective perceptions.

Taken together, this is the first systematic review that shows the impact of COVID-19 on mental health among adolescents. Hence, this study might be important in the aspect of a more holistic approach towards adolescents who are affected by COVID-19, whether directly or indirectly. In the long term, there might be a surging incidence and prevalence of mental health disorders which might be attributed to COVID-19 and hence awareness and interventions are needed which might require cooperations between the adolescents, families, medical care workers, and the governments.

## Conclusion

COVID-19 has been found to be associated with adolescents mental health changes, especially the fear of COVID-19 in a population with adequate exposure of COVID-19 was proved to create adverse mental health condition such as anxiety and depression. adolescents who had experienced previous trauma with addition of social isolation/quarantine and loneliness were more prone towards anxiety and depression during and even after the enforced isolation ends. On the other hand, some protective factors were found to help adolescents stay away from any mental health adverse impacts due to COVID-19. Physical-psychosocial support provision, adequate and accurate information from credible source about COVID-19, and good motivation to obey physical distancing has shown to decrease the likelihood of negative mental health changes in adolescent. Through this systematic review, psychiatrist, pediatrician, parents, or other parties who accompany or take care of adolescents hopefully can raise awareness to detect mental health changes in order to decrease adverse mental health impacts in adolescent’s future. Further study needs to be done to find other factors which may be associated to mental health changes besides fear of COVID-19 and social distancing.

## Data Availability

Available upon request

## Abbreviations

ASD: Autism spectrum disorder
CDC: Centers for Disease Control
COVID-19: Coronavirus disease-19
GAD: Generalized Anxiety Disorder Scale
MSN: Media Social Networking
NOS: Newcastle Ottawa Scale
OCD: Obsessive compulsive disorder
PHQ: Patient Health Questionnaire
PRISMA: Preferred Reporting Items for Systematic Reiew and Meta-Analysis
PROSPERO: The International Prospective Register of Systematic Reviews
PSM: Propensity Score Matching
PTSD: Post-traumatic stress disorder
SLSS: Social Live Streaming Services
WHO: World Health Organization
